# Analysis of Human Brain Structure Reveals that the Brain “Types” Typical of Males Are Also Typical of Females, and Vice Versa

**DOI:** 10.3389/fnhum.2018.00399

**Published:** 2018-10-18

**Authors:** Daphna Joel, Ariel Persico, Moshe Salhov, Zohar Berman, Sabine Oligschläger, Isaac Meilijson, Amir Averbuch

**Affiliations:** ^1^School of Psychological Sciences, Tel Aviv University, Tel Aviv, Israel; ^2^Sagol School of Neuroscience, Tel Aviv University, Tel Aviv, Israel; ^3^School of Computer Science, Tel Aviv University, Tel Aviv, Israel; ^4^Max Planck Research Group for Neuroanatomy and Connectivity, Max Planck Institute for Human Cognitive and Brain Sciences, Leipzig, Germany; ^5^Faculty of Life Sciences, University Leipzig, Leipzig, Germany; ^6^International Max Planck Research School NeuroCom, Leipzig, Germany; ^7^School of Mathematical Sciences, Tel Aviv University, Tel Aviv, Israel

**Keywords:** sex differences, gender differences, brain, MRI, female brain, male brain

## Abstract

Findings of average differences between females and males in the structure of specific brain regions are often interpreted as indicating that the typical male brain is different from the typical female brain. An alternative interpretation is that the brain types typical of females are also typical of males, and sex differences exist only in the frequency of rare brain types. Here we contrasted the two hypotheses by analyzing the structure of 2176 human brains using three analytical approaches. An anomaly detection analysis showed that brains from females are almost as likely to be classified as “normal male brains,” as brains from males are, and vice versa. Unsupervised clustering algorithms revealed that common brain “types” are similarly common in females and in males and that a male and a female are almost as likely to have the same brain “type” as two females or two males are. Large sex differences were found only in the frequency of some rare brain “types.” Last, supervised clustering algorithms revealed that the brain “type(s)” typical of one sex category in one sample could be typical of the other sex category in another sample. The present findings demonstrate that even when similarity and difference are defined mathematically, ignoring biological or functional relevance, sex category (i.e., whether one is female or male), is not a major predictor of the variability of human brain structure. Rather, the brain types typical of females are also typical of males, and vice versa, and large sex differences are found only in the prevalence of some rare brain types. We discuss the implications of these findings to studies of the structure and function of the human brain.

## Introduction

Findings of average differences between females and males in the structure and function of specific brain regions as well as evidence from *in vitro* and *in vivo* studies that sex can affect the structure and function of brain cells are often interpreted as indicating that the typical male brain is different from the typical female brain (e.g., Baron-Cohen, 2002; Ingalhalikar et al., 2014; Larson et al., 2015; Ecker et al., 2017; Wierenga et al., 2017). At its extreme, the interpretation is that brains from females and from males belong to two distinct categories, just as male and female genitals are. This interpretation is very common in popular discussions of sex and the brain (e.g., Sax, 2005; Brizendine, 2006), but can also be found in scientific publications, as in the following statements: “males and females are biologically different not only with regards to gonads and secondary sexual characteristics but also in the structure and, more importantly, the function of many other organs including the brain” (Grgurevic and Majdic, 2016, p. 1481), and “sex-specific differences in dopaminergic, serotonergic, and gamma-aminobutyric acid (GABA)ergic markers indicate that male and female brains are neurochemically distinct” (Cosgrove et al., 2007, p. 847). The less extreme interpretation, which is more common in scientific publications and which is the one challenged in the present study, is that although there is overlap between females and males in brain structure, the typical female brain differs from the typical male brain. This is evident in describing average group-level differences between females and males as if they were characteristics of females and males, or in assuming that human brains are aligned along a continuum between a typical male brain and a typical female brain. The former is evident in statements such as: “During developmental periods, male brains tend to be structured to facilitate within-lobe and within-hemisphere connectivity …In contrast, female brains tend to have better interhemispheric connectivity and better cross-hemispheric participation…” (Tyan et al., 2017, p. 380). The latter may be seen in the description of the aim of a recent study: “to examine the probability of autism spectrum disorder along a normative phenotypic axis ranging from the characteristic female to male brain phenotype” (Ecker et al., 2017, p. 330).

In contrast, one of us (Joel, 2011, 2012; Joel et al., 2015; Joel and Fausto-Sterling, 2016) has claimed that group-level sex differences in specific brain features do not “add-up” to create two types of brains, one typical of females and the other typical of males, but rather that what is typical of both males and females is a brain comprised of a “mosaic” of features, some in the form more common in males and some in the form more common in females. Under this scheme, the brain types typical of females are also typical of males and vice versa, but there are sex differences in the frequency of rare brain mosaics. For example, brains comprised of only features with the form that is more common in males than in females are rare in the population, but of the people with such brains, there are more males than females (Joel and Fausto-Sterling, 2016).

The present study used two analytical approaches, new in this context, to contrast the two hypotheses – the hypothesis that the typical female brain is different from the typical male brain and the hypothesis that the brain types typical or females are also typical of males, but differences exist in the frequency of rare brain types. The first analytical approach used an anomaly detection algorithm to test whether the “types” of brain typical of females are also typical of males, and vice versa. Anomaly detection aims to build a model of “normal” items so that it can detect an “abnormal” item when it appears, without having a priori knowledge on the characteristics of the “abnormal” item or on what distinguishes it from the “normal” items. In this sense it is unsupervised learning. Here, an anomaly detection algorithm was applied to examples of brains from a single sex category (say, females) to create a model of brains of this sex category, and then the model was used to identify for every new brain (i.e., from females who were not included in the training set and from males) whether it belongs to this group of brains (“normal”) or does not (“anomalous”). Next, the exact same analysis was repeated, but this time using brains from the other sex category (i.e., males) to create the model. If the brain types typical of males are also typical of females, similar proportions of females and males are expected to be labeled as “normal” in the test stage, regardless of the sex category of the brains used to create the model. In contrast, if the brain types typical of males differ from the brain types typical of females, more females than males are expected to be labeled as “normal” following training on brains from females, and more males than females are expected to be labeled as “normal” following training on brains from males.

Anomaly detection can only answer whether the brain types typical of one sex category are also typical of the other sex category, and vice versa. To answer whether there are large sex differences in the prevalence of rare (i.e., “anomalous”) brain types we complemented the anomaly detection analysis with unsupervised clustering. Two algorithms were used to find clusters that best describe variability in a population of human brains regardless of sex category. Each algorithm was run nine times, to create between 2 and 10 clusters, and the proportion of males and females within each cluster was assessed. Assuming that each cluster represents a brain “type,” if the brain types typical of females are also typical of males, similar proportions of females and males are expected in the large clusters – that is, in the brain types typical of humans, but different proportions of females and males are expected in some of the small clusters – that is, in some of the rare brain types. In contrast, if the brain types typical of females are different from the brain types typical of males, some large clusters are also expected to show large sex differences in the proportion of females and males, with some clusters being predominantly female, and others predominantly male.

While a failure to find large sex differences in the proportion of females and males in the large clusters indicates that sex category is less important than other variables in determining brain structure, it does not indicate that brains cannot be clustered according to sex category. As has been previously argued (Joel, 2011) and demonstrated (Chekroud et al., 2016; Del Giudice et al., 2016; Joel et al., 2016; Rosenblatt, 2016), the existence of group-level sex differences in the structure of specific brain regions suffices for predicting, with accuracy above chance, whether a brain’s owner is male or female. In this sense, brains can be classified as “male” and “female” (Chekroud et al., 2016; Del Giudice et al., 2016; Rosenblatt, 2016). The question is whether this classification indeed captures a core difference between human females and males, or rather is specific to the subpopulation of humans on which the classification model was built. With this question in mind, two algorithms of supervised clustering were applied to find the two clusters which best separate brains from females and brains from males in four subpopulations, each from a different geographical region. We then tested whether the brains considered typical of males and females in one subpopulation were also typical of males and females in other subpopulations.

In all parts of the study, in order to increase the generalizability of our conclusions we used two datasets of magnetic resonance images of human brains, analyzed with two methods (volume- and surface-based analysis), as well as different linear and non-linear dimensionality reduction transformations of the information extracted by these methods. In all analyses, the different methods were applied to all the data available in a dataset [this is in contrast to Joel et al. (2015) where only regions showing the largest sex differences were included in the analysis].

We would like to note that all the analytical approaches applied in the present study treat similarity and difference in a mathematical sense and not in a biological sense. Thus, it is not known, and has not been tested in the present study, whether and in what biological sense (structurally or functionally) a brain is more similar to other brains that are similarly classified than to brains from another classification/cluster. In fact, we claim here (see the section “Discussion”) and elsewhere (e.g., Joel et al., 2016) that the mosaic nature of the brain makes such classifications functionally meaningless.

We would also like to note that we do not attempt to disentangle the effects of sex from the effects of gender – the set of psychological and environmental variables that correlate with sex (e.g., socioeconomic status, type of education, and personality characteristics; Fausto-Sterling, 2000; Fine, 2010; Rippon et al., 2014; Joel and Fausto-Sterling, 2016; Joel and McCarthy, 2016; Maney, 2016). In the present study we ignore the probable effects of gender on observed differences between females and males in brain structure, as we ask whether these differences, regardless of their cause (sex, gender, their interactions), “add up” to create two distinct brain types, one typical of males and the other typical of females (for a mathematical illustration of this problem, see Joel and Fausto-Sterling, 2016). We use “sex” and not “gender” throughout the text, as the measure obtained in the different datasets and used here for analysis is sex category (female, male) as marked by participants, and not measures of gender (e.g., gender identity and gender role).

## Materials and Methods

### Data Collection and Preparation for Analysis

#### Imaging Data

Data were obtained from three sources: Tel-Aviv University and the 1000 Functional Connectomes Project (Biswal et al., 2010), which were combined into a single sample named Connectomes+ (this dataset can be found online^1^), and the Brain Genomics Superstruct Project (GSP) Open Access Data Release (Holmes et al., 2015). For details of the imaging protocols and the datasets included from the 1000 Functional Connectomes Project see Joel et al. (2015).

#### Volume-Based Analysis

Images were analyzed using MATLAB (MathWorks, Natick, MA, United States) and SPM8 (Wellcome Department of Cognitive Neurology, London, United Kingdom^2^). Gray matter volume was assessed with the optimized voxel-based morphometry (VBM) protocol (Good et al., 2001), using the standard segmentation and registration tools available in the software. Images were normalized, segmented, modulated, and smoothed with an 8-mm Gaussian kernel. Voxels were mapped into 116 regions according to the Automated Anatomical Labeling (AAL) atlas (Tzourio-Mazoyer et al., 2002, **Supplementary Appendix I**), and mean gray matter volume was calculated for each region for each participant.

#### Surface-Based Analysis

The FreeSurfer software package (Athinoula A. Martinos Center for Biomedical Imaging, Harvard University, Cambridge, MA, United States^3^) was used to generate the surface representations of the cortex and to delineate 68 regions (see **Supplementary Appendix II** for the full list of regions). For each participant we calculated the average cortical thickness (gray–white matter boundary to pial boundary; Fischl et al., 2004) and total cortical volume for each of these regions, as well as the volumes of 77 white matter regions and of 23 subcortical structures (**Supplementary Appendix II**). In addition, we calculated the “corrected” volumes of these 168 regions using the power-proportion method (Liu et al., 2014). (For review and discussion of the controversy regarding the “right” way to take individual differences in total brain volume into account see, for example, Liu et al., 2014; Pintzka et al., 2015; Snoek et al., 2018).

#### Additional Datasets

To validate our approach, we also applied the anomaly detection and the unsupervised clustering algorithms to the facial morphology of two primate species and to highly gender-stereotyped behaviors in university students.

#### Primates’ Faces

The *x, y*, and *z* coordinates of 20 landmarks located on the face of 90 monkeys (31 *Cebus apella* and 59 *Macaca fascicularis*) were kindly provided by Corner and Richtsmeier (1991) and Richtsmeier et al. (1993a,b). These data were corrected for inter-species differences in skull size and analyzed to yield 190 distances between 20 facial landmarks (Del Giudice et al., 2016). These 190 distances were used in all subsequent analyses.

#### Gender-Stereotyped Behaviors (Carothers and Reis, 2013)

Data were obtained from Harry Reis. The data consisted of 10 highly gender-stereotyped activities (boxing, construction, playing golf, playing video games, scrapbooking, taking a bath, talking on the phone, watching porn, watching talk shows, cosmetics) of 263 students (106 men, 157 women) from an introductory-level psychology class at a large Midwestern American University. These activities were specifically selected to differentiate between men and women of this specific culture (all |Cohen’s *d*| > 1.00, Carothers and Reis, 2013). Applying taxometric methods to these data yielded “male” and “female” classes, each containing about 90–93% of the students from the corresponding sex category (Figure 2d in Carothers and Reis, 2013).

In all datasets, in order not to bias the analysis due to unequal number of females and males, we randomly selected females so that the number of females and males would be equal.

### Dimensionality Reduction

Due to dependencies, data points in high-dimensional big data usually reside in a lower dimensional subspace. Here, in addition to analyzing the data in the original space (following a *z*-score transformation), both linear and non-linear dimensionality reduction methods were applied.

#### Principal Component Analysis (PCA)

Principal component analysis is a linear dimensionality reduction method, in which distance between subjects (e.g., brains) in the low-dimension space represents the distance between subjects on the most significant principal components of the original data. The first principal component explains the largest possible variance (eigenvalue) and each succeeding component explains the highest variance possible under the constraint of orthogonality to the preceding components (Jolliffe, 1986; Hotelling, 1993). The dimension of the low-dimensional space (i.e., the number of principal components used) is determined by sorting the eigenvalues and finding the smallest number of eigenvalues that incorporate most of the variance, according to some criterion (Halko et al., 2011; Aizenbud et al., 2016). The cutoff applied here is the common “elbow” (the scree test, Cattell, 1966), the number of components where the cumulative sum of eigenvalues has unit slope. Although this and other thresholding methods have been improved by studying asymptotics of the plot of the sorted principal components (e.g., Cangelosi and Goriely, 2007; Gavish and Donoho, 2014), our data display a clear break on all occasions, so the choice of hard threshold is not critical.

#### Diffusion Mapping (DM)

Diffusion mapping is a non-linear dimensionality reduction method, in which distance between points in the low-dimension (embedded) space represents the diffusion distances of the original data (Coifman and Lafon, 2006). DM was performed as previously described (Salhov et al., 2015), with (a required parameter) 𝜀 set as the third power of the mean value of the data. To preserve distances in the embedded space, three approximations of the DM embedding were used: Isometric DM (μIDM, Salhov et al., 2015) and incomplete pivoted QR decomposition with (ICPQR) and without the DM kernel (ICPQRd, Salhov et al., 2015; Bermanis et al., 2016). The first two were used with 𝜀 set as in DM and several values of (another required parameter) μ (10^-8^, 10^-6^, 10^-4^, and 10^-2^). ICPQRd was used with the same values of μ as for μIDM and ICPQR.

### Anomaly Detection

Anomaly detection refers to a process that identifies in a given dataset patterns that do not conform to established or expected “normal” behavior. In the training step, the average Euclidean distance between every data point in the training set and its *k* nearest neighbors was calculated. In the detection step, the average Euclidean distance between the new data point and its *k* nearest neighbors was calculated, and if it was larger than a threshold, the data point was classified as “abnormal.” In the present study, half of the runs were carried out with brains from females as the training set, and half with brains from males as the training set. In each run, half of the brains from the training sex category were used as the training set and the other half of the brains from this sex category as well as half of the brains from the other sex category were used as the test set. The algorithm was applied 72 times for each dataset, with nine *k*-values (10, 15, 20, …, 50) and eight thresholds (40%, 45%, 50%, …, 75%; note that the higher the threshold, the higher the classification of data points as “normal”) (David, 2009).

### Unsupervised Clustering

#### K-Means

This method partitions the observations into *k* clusters chosen so as to minimize the within-cluster sum of squares. Each observation is assigned to the cluster with the nearest mean (Lloyd, 1957). The initial clusters were randomly chosen using the method described in Jongen et al. (2009).

#### Hierarchical

This method clusters data points on the basis of the local geometry of the data. It starts with each data point being a cluster, and in each step merges two clusters into one (whence the name hierarchical clustering) until a desired number of clusters is reached (David and Averbuch, 2012). The Ward linkage method was used to choose the two clusters that should be merged in each step (Ward, 1963).

### Modeling the Dependence of Sex Disparity on Cluster Size

To test the hypothesis that a disparity in the proportion of females and males in a cluster depends on cluster size, we took sex disparity as *P* = max(*q*, 1-*q*), where *q* is the proportion of females in the cluster. Assuming that a cluster of size *n* chooses *P* from a distribution with mean *p*(*n*) and standard deviation *s*(*n*), the data obtained by the two clustering algorithms were used to estimate these two functions, as smooth functions of cluster size (**Supplementary Appendix III**).

### Supervised Clustering

#### Support Vector Machine (SVM)

Support vector machine training algorithms (Vapnik, 1995) use a set of training examples, each marked as belonging to one of two categories (e.g., male and female), to build a model that assigns new examples into one of the categories. The version applied here used SVM with linear kernel based on sequential minimal optimization algorithm (Platt, 1998).

#### Random Forests

Random forests (Ho, 1995; Breiman, 2001) is a supervised learning method for classification that is based on the “divide and conquer” principle. It avoids overfitting by aggregating multiple decision trees.

### Software

Analyses were performed with Matlab R2017a, using the toolboxes: “MATLAB” – v9.2, “System Identification Toolbox” – v9.6, “Statistics and Machine Learning Toolbox” – v11.1, “Curve Fitting Toolbox” – v3.5.5, “Bioinformatics Toolbox” – v4.8, “Parallel Computing Toolbox” – 6.10, and “MATLAB Distributed Computing Server” – v6.10. The code can be found online^4^.

## Results

### Anomaly Detection: Are the Brain Type(s) Typical of Females Also Typical of Males, and Vice Versa?

**FIGURE 1:**
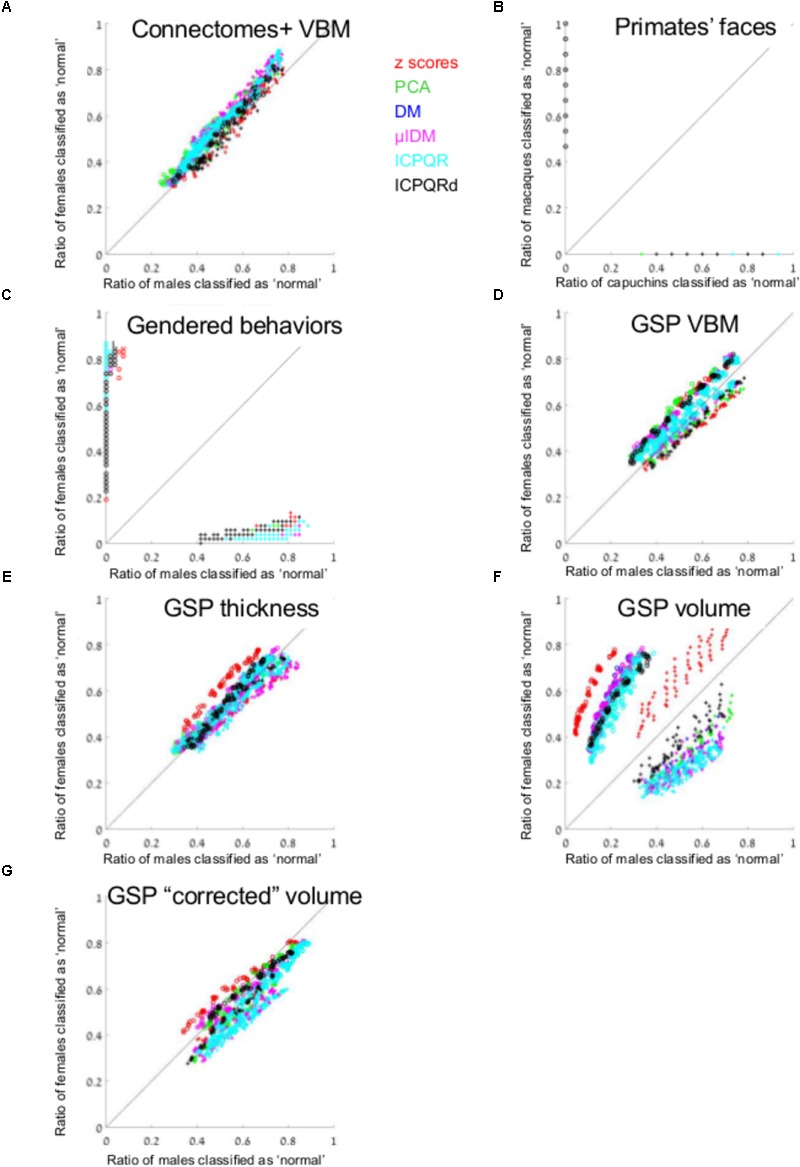
Anomaly detection. **(A, C–G)** The percent of males (out of males, *X*-axis) and the percent of females (out of females, *Y*-axis) that were classified as “normal,” when the model was built on males (pluses) and when it was built on females (circles) from **(A)** Connectomes+ VBM data, **(C)** Carothers and Reis’ behavioral data, **(D)** GSP VBM data, **(E)** GSP cortical thickness data, **(F)** GSP volume data, **(G)** GSP ICV-“corrected” volume data. **(B)** The percent of capuchins (out of capuchins, *X*-axis) and the percent of macaques (out of macaques, *Y*-axis) that were classified as “normal,” when the model was built on macaques (circles) and when it was built on capuchins (pluses). The results from each dimension reduction method are marked with a different color. Across all brain-related datasets presented in the figure, the number of dimensions that were included in the analysis ranged between 2 and 94, median = 13. DM, diffusion mapping, Euclidean distances; ICPQR, incomplete pivoted QR decomposition with the kernel of the diffusion map; ICPQRd, incomplete pivoted QR decomposition without the kernel of the diffusion map; μIDM, isometric diffusion map; PCA, principle component analysis.

We used the gray matter volume of 116 regions defined using VBM of 466 females and 466 males from Joel et al.’s (2015) study (Connectomes+). **Figure 1A** presents the percent of males (out of males, *X*-axis) who were classified as “normal” [i.e., correct classification when the model was built on males (pluses) and incorrect classification when it was built on females (circles)], and the percent of females (out of females, *Y*-axis) who were classified as “normal,” for each of the 864 runs of the algorithm [once with brains from females as the training set (circles) and once with brains from males (pluses) × six data transformation methods × nine *k*-values × eight threshold values]. As can be seen, the percent of females classified as “normal” when the algorithm was trained on brains from males was very similar to the percent of males classified as “normal” following such training, and vice versa (compare the distribution of the pluses and circles to the *y* = *x* black line). In fact, on average, brains from females were 1.09 more likely to be classified as “normal” than brains from males were, when training was carried out on brains from females, and 1.07 more likely to be classified as “normal” than brains from males were, when training was carried out on brains from males.

For comparison, all the applications of the anomaly detection algorithm to the facial morphology dataset detected all the faces from the species they were not trained on, as anomalous (**Figure 1B**). Applying the anomaly detection algorithm to the gender-stereotyped behaviors dataset revealed that females were at least 31 more likely to be classified as “normal” than males were, when training was carried out on females, and males were at least 22 more likely to be classified as “normal” than females were, when training was carried out on males (at least, because these are the mean ratios over the non-zero “normal” cases, **Figure 1C**).

We next repeated the same analysis on the data of 622 females and 622 males obtained from the Brain GSP. This dataset was more homogenous than the first dataset in terms of age (18–35 years of age compared with 18–79 years), geographical region (all participants underwent scanning in Boston, United States, compared with scanning in Tel-Aviv, Beijing, Cambridge, and other locations), and imaging parameters (a single imaging protocol compared with different protocols in different imaging sites). The results obtained with the GSP dataset were similar to those obtained with the Connectomes+ dataset in that on average, brains from females were 1.16 more likely to be classified as “normal” than brains from males were, when training was carried out on brains from females, and brains from males were 1.05 more likely to be classified as “normal” than brains from females were, when training was carried out on brains from males (**Figure 1D**).

To test whether the pattern of results obtained in the two brain imaging datasets was dependent on the type of analysis of the imaging data (VBM), we performed the same analysis on a subgroup of the GSP sample (559 females, 559 males), whose T1-weighted images were preprocessed for cortical surface-based analysis. Analysis of the cortical thickness of 68 cortical regions yielded very similar results to those obtained with the VBM analysis of these data (**Figure 1E** and **Table 1**).

**Table 1:** Details of datasets and summary of main findings.

	Number of characteristics and subjects	Anomaly detection	Unsupervised clustering K-means	Unsupervised clustering hierarchical
Primate faces	190 Capuchin, 31 Macaque, 31	^∗^	C&Mc: 0.00 (0.00) C&C: 1.00 (1.00) Mc&Mc: 1.00 (1.00)	C&Mc: 0.00 (0.00) C&C: 1.00 (1.00) Mc&Mc: 1.00 (1.00)
Gendered behaviors	10 Female, 101 Male, 101	“Male” model: 22^∗∗^ “Female” model: 31^∗∗^	F&M: 0.12 (0.14) F&F: 0.81 (0.77) M&M: 0.96 (0.98)	F&M: 0.08 (0.13) F&F: 0.86 (0.82) M&M: 0.98 (0.95)
Connectomes+ VBM	116 Female, 466 Male, 466	“Male” model: 0.93 “Female” model: 1.09	F&M: 0.52 (0.52) F&F: 0.55 (0.55) M&M: 0.51 (0.51)	F&M: 0.68 (0.59) F&F: 0.74 (0.62) M&M: 0.64 (0.58)
GSP VBM	116 Female, 622 Male, 622	“Male” model: 1.05 “Female” model: 1.16	F&M: 0.49 (0.49) F&F: 0.54 (0.53) M&M: 0.50 (0.50)	F&M: 0.48 (0.55) F&F: 0.53 (0.56) M&M: 0.51 (0.59)
GSP, SBA, cortical thickness	68 Female, 559 Male, 559	“Male” model: 1.05 “Female” model: 1.06	F&M: 0.50 (0.50) F&F: 0.50 (0.50) M&M: 51 (51)	F&M: 0.61 (0.55) F&F: 0.67 (0.57) M&M: 0.57 (0.55)
GSP, SBA, volume	168 Female, 559 Male, 559	“Male” model: 1.68 “Female” model: 3.05	F&M: 0.35 (0.35) F&F: 0.71 (0.71) M&M: 0.61 (0.61)	F&M: 0.37 (0.42) F&F: 0.73 (0.72) M&M: 0.56 (0.64)
GSP, SBA, volume, split by brain size	168 Female, 559 Male, 559		S&L: 0.19 (0.19) S&S: 0.89 (0.89) L&L: 0.75 (0.75)	S&L: 0.26 (0.34) S&S: 0.94 (0.82) L&L: 0.63 (0.71)
GSP, SBA, “corrected” volume	168 Female, 559 Male, 559	“Male” model: 1.23 “Female” model: 0.96	F&M: 0.51 (0.51) F&F: 0.51 (0.51) M&M: 0.53 (0.53)	F&M: 0.60 (0.62) F&F: 0.56 (0.59) M&M: 0.67 (0.67)

*Anomaly detection: The mean ratio of the classification rate as “normal” for the group on which the model was built and for the other group. Unsupervised clustering: Chances to be in the same cluster, in the best separating case and on average (in brackets). ^∗^Was not calculated because the classification rate as “normal” for the other species was always zero. ^∗∗^Mean over the non-zero “normal” cases. C, capuchin; F, female; L, large; M, male; Mc, macaque; S, small.*

Last, we analyzed the “uncorrected” volume (rather than cortical thickness) of the 68 cortical regions as well as of 23 subcortical gray matter regions and 77 white matter regions (**Figure 1F** and **Table 1**). This analysis yielded a somewhat different pattern of results, especially when brains from females were used as the model, with brains from females being on average 3.05 more likely to be classified as “normal” than brains from males were. (When training was carried out on brains from males, brains from males were 1.68 more likely to be classified as “normal” than brains from females were.) Because previous studies found that observed sex differences in the brain are largely attributed to differences in brain size (e.g., Im et al., 2008; Hänggi et al., 2014; Jäncke et al., 2015; Coupe et al., 2017), we repeated the same analysis on the volume of the 168 gray and white matter regions after “correcting” for differences in brain volume using the power-proportion method (Liu et al., 2014). This analysis revealed that similar proportions of females and males were labeled as “normal,” regardless of the sex category used for training (**Figure 1G** and **Table 1**).

### Unsupervised Cluster Analysis: Are There Similar or Different Proportions of Females and Males in “Typical” and “Rare” Human Brain “Types”?

#### Divisions Into Two Clusters

**FIGURE 2:**
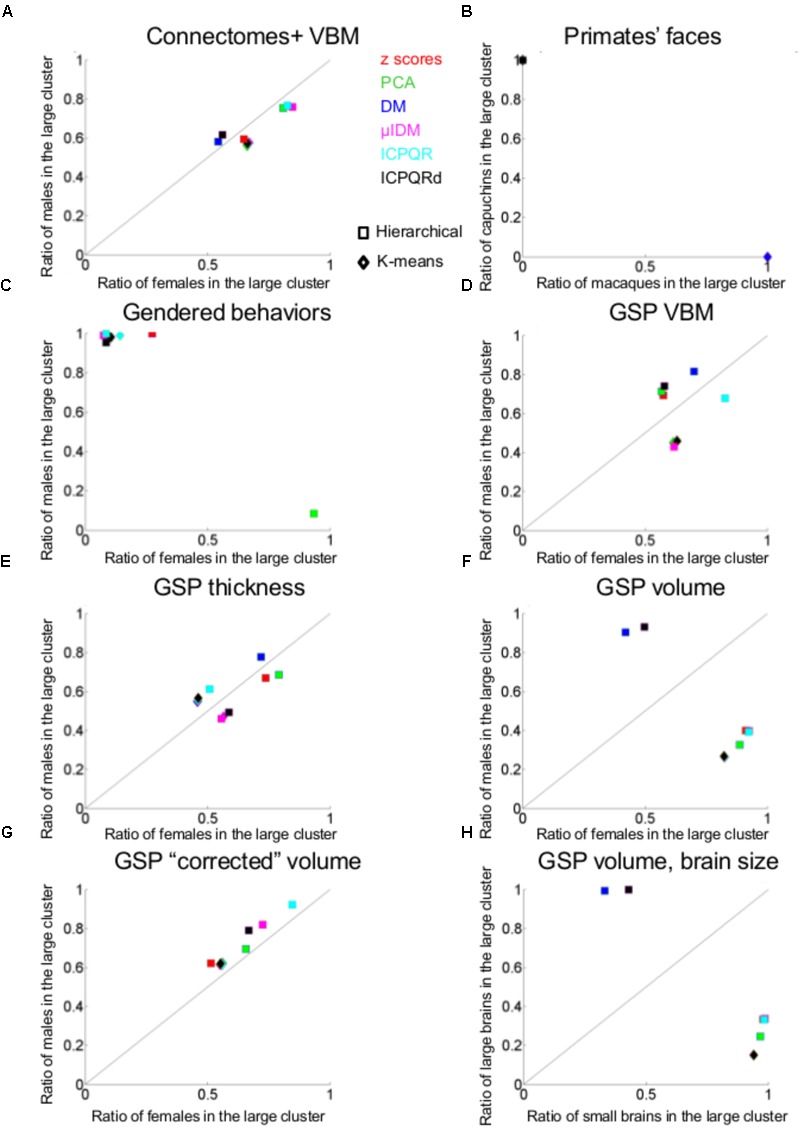
Unsupervised clustering: dividing into two clusters. **(A, C–F, H)** The percent of females (out of females, *X*-axis) and the percent of males (out of males, *Y*-axis) that were included in the larger clusters in each of the divisions into two clusters by the hierarchical (squares) and *k*-means (rhombuses) clustering algorithms of **(A)** Connectomes+ VBM data, **(C)** Carothers and Reis’ behavioral data, **(D)** GSP VBM data, **(E)** GSP cortical thickness data, **(F)** GSP volume data, **(H)** GSP ICV-“corrected” volume data. **(B)** The percent of macaques (out of macaques, *X*-axis) and the percent of capuchins (out of capuchins, *Y*-axis) that were included in one of the clusters in each of the seven divisions into two clusters by the hierarchical (squares) and *k*-means (rhombuses) clustering algorithms. **(G)** The percent of small brains (out of small brains, *X*-axis) and the percent of large brains (out of large brains, *Y*-axis) that were included in the larger cluster in each of the divisions into two clusters by the hierarchical (squares) and *k*-means (rhombuses) clustering algorithms. DM, diffusion mapping, Euclidean distances; ICPQR, incomplete pivoted QR decomposition with the kernel of the diffusion map; ICPQRd, incomplete pivoted QR decomposition without the kernel of the diffusion map; μIDM, isometric diffusion map; PCA, principle component analysis.

**Figure 2A** presents the results of applying the hierarchical (squares) and *k*-means (rhombuses) clustering algorithms to the Connectomes+ dataset. The figure presents for each division the percent of females (out of females) and the percent of males (out of males) contained in the larger of the two clusters. All the divisions yielded a large cluster that contained over half of the females (55–85%) and over half of the males (58–76%), with the proportions of females and males within each cluster being very similar (compare the distribution of the squares and rhombuses to the *y* = *x* black line). The division showing the largest difference (i.e., the most separating division) yielded a cluster containing 66% of the females and 56% of the males. This means that under the greatest separation between females and males, the chances for a male and a female to be in the same cluster were 52%, compared to 55 and 51%, which were the chances, respectively, that two females or two males would be in the same cluster (see **Table 1** for these chances in the best separating case and on average, for each clustering algorithm separately).

For comparison, when cluster analysis was applied to the primate facial morphology data (**Figure 2B**), under all divisions, the chances that a macaque and a capuchin would be in the same cluster were 0%, compared to a 100% chance that two macaques or two capuchins would be in the same cluster (**Table 1**). When cluster analysis was applied to the gender-stereotyped behaviors (**Figure 2C**), the chances that a male and a female would be in the same cluster under the best separating division were 12%, compared to 81 and 96%, which were the chances, respectively, that two females or two males would be in the same cluster (**Table 1**).

Cluster analysis of the GSP-VBM and GSP-cortical thickness datasets yielded similar results to those obtained with the Connectomes+ dataset, in that the proportions of females and males within a single cluster were quite similar regardless of the clustering algorithm being used or the type of data transformation (**Figures 2D,E**). As a result, the chances that a female and a male would be in the same cluster were similar to the chances that two females or two males would be in the same cluster (**Table 1**). However, in contrast to the Connectomes+ dataset in which in all divisions the larger cluster contained most of the females and most of the males, this was true for only some of the divisions of the GSP datasets (all of which were created by the hierarchical algorithm). In the remaining divisions, one cluster contained most of the females and the other cluster contained most of the males.

A different pattern of results was obtained when we analyzed the “uncorrected” volume of 168 gray and white matter regions. All the divisions yielded a large cluster containing most (82–93%) of the subjects of one sex category and a medium to large minority (26–50%) of the subjects from the other sex category (**Figure 2F** and **Table 1**). Under the best separating division, the chances for a male and a female to be in the same cluster were 35%, compared to 71 and 61%, which were the chances, respectively, that two females or two males would be in the same cluster. Yet, repeating the same analysis on the “corrected” volumes revealed that all the divisions yielded a large cluster that contained over half of the females (51–84%) and over half of the males (62–92%), with very similar proportions of females and males in each cluster (**Figure 2G** and **Table 1**). This latter result suggests that, when applied to “uncorrected” volumes, the algorithms were dividing brains into large and small rather than into male and female. To further test this possibility, we assessed the composition of the clusters obtained when the “uncorrected” data were used, in terms of large versus small brains [defined, respectively, as above and below the median of the intracranial volume (ICV); with this definition, 83% of the females had a small brain and 83% of the males had a large brain]. Indeed, this analysis revealed that the large cluster contained 94–100% of the small (or large) brains and between 15 and 42% of the large (or small) brains (**Figure 2H** and **Table 1**).

#### Divisions Into 2–10 Clusters

**FIGURE 3:**
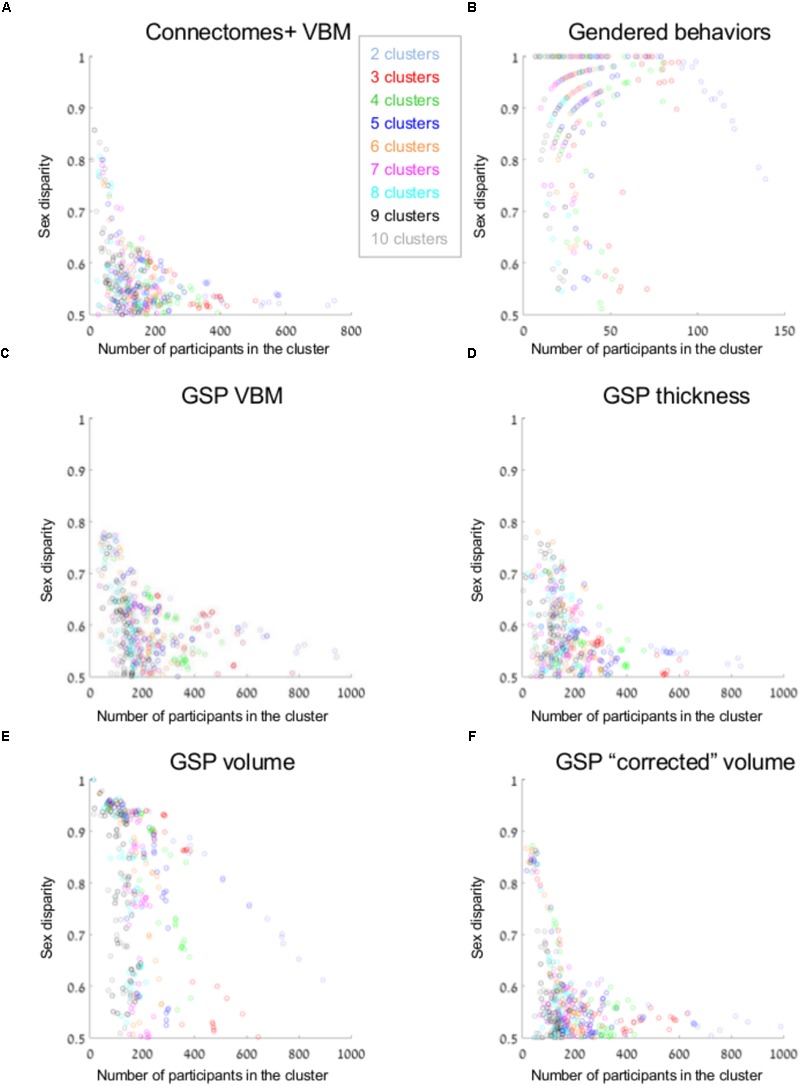
Unsupervised clustering: dividing into 2–10 clusters. **(A–F)** The sex disparity (i.e., the largest of the proportion of females and the proportion of males in a cluster, *Y*-axis) as a function of cluster’s size (*X*-axis) of every cluster, following divisions into 2–10 clusters (the number of clusters is marked with different colors) of the **(A)** Connectomes+ VBM data, **(B)** Carothers and Reis’ behavioral data, **(C)** GSP VBM data, **(D)** GSP cortical thickness data, **(E)** GSP volume data, and **(F)** GSP ICV-“corrected” volume data.

**FIGURE 4:**
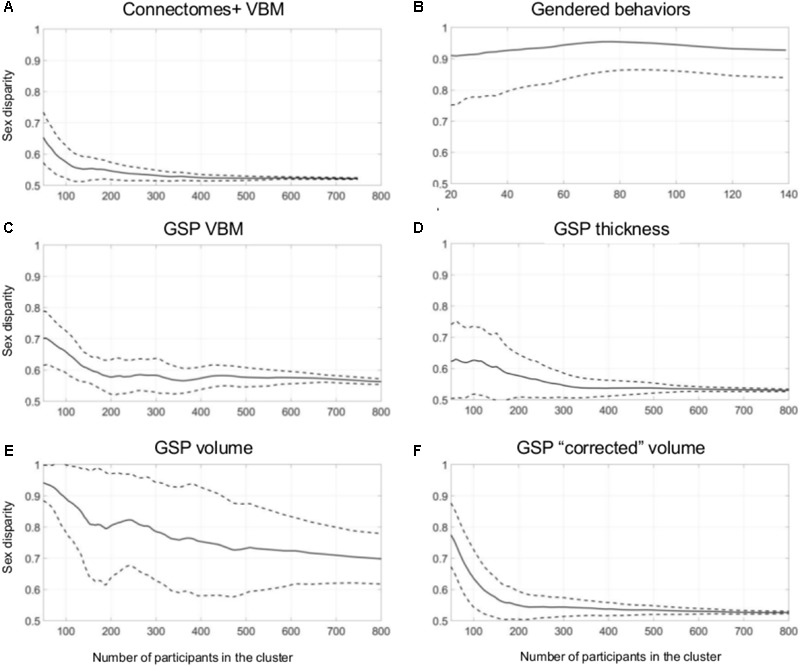
Unsupervised clustering: dividing into 2–10 clusters. **(A–F)** The estimated sex disparity (*P, Y*-axis) as a function of cluster size (*X*-axis), following division into 2–10 clusters of the **(A)** Connectomes+ VBM data, **(B)** Carothers and Reis’ behavioral data, **(C)** GSP VBM data, **(D)** GSP cortical thickness data, **(E)** GSP volume data, **(F)** GSP ICV-“corrected” volume data. The solid curve presents the mean *P*, and the two dashed curves present mean *P* ±1 standard deviation.

**Figure 3A** presents sex disparity (i.e., the larger of the proportion of males and the proportion of females, *Y*-axis) in a cluster, as a function of the cluster’s size (*X*-axis), following divisions of the Connectomes+ VBM dataset into 2–10 clusters (clusters obtained following division into the same number of clusters are painted in the same color; note that the figure depicts all the clusters that were created by the two clustering algorithms following the different dimension reduction methods). As can be seen, the variability in sex disparity in the very small clusters (less than 100 brains) was very high, with no sex differences (i.e., sex disparity close to 0.5) in some clusters and very large sex differences (up to approximately six times more brains from one sex category) in other clusters. The variability in sex disparity dropped dramatically as the cluster size increased, with very similar proportions of females and males in clusters with more than 300 brains (the dataset included 936 brains). We next applied a mathematical model to identify the mean and standard deviation of sex disparity (*P*) as a function of cluster size (**Figure 4A**). As can be seen, the mean and variability of *P* dropped quickly with increasing cluster size, and stabilized around 0.52 with very little variability (the central curve presents the mean *P*, and the other two curves present mean *P* ±1 standard deviation). For comparison, a similar analysis of the gendered behaviors revealed that most clusters, irrespective of size, showed a large difference in the proportion of females and males in the cluster, with *P* around 0.9 (**Figures 3B, 4B**).

Last, we calculated for every division the chances that a female and a male would be in the same cluster and the chances that two males or two females would be in the same cluster. As was the case with divisions into two clusters, these chances were very similar also following divisions into 3–10 clusters (**Table 2**).

**Table 2:** Mean (SD) chances to be in the same cluster, for 3–6 and 7–10 divisions.

		Mean (SD) chances to be in the same cluster, for 3–6 divisions							
**Number of clusters:**		**3**		**4**		**5**		**6**	
		**K**	**H**	**K**	**H**	**K**	**H**	**K**	**H**

Connectomes+ VBM	F&M	0.36 (0.003)	0.38 (0.042)	0.27 (0.002)	0.27 (0.020)	0.22 (0.014)	0.24 (0.015)	0.18 (0.012)	0.20 (0.018)
	F&F	0.38 (0.005)	0.40 (0.047)	0.28 (0.007)	0.28 (0.022)	0.23 (0.016)	0.26 (0.016)	0.20 (0.018)	0.22 (0.019)
	M&M	0.35 (0.002)	0.37 (0.037)	0.26 (0.002)	0.27 (0.019)	0.21 (0.009)	0.24 (0.014)	0.18 (0.006)	0.20 (0.016)
GSP VBM	F&M	0.34 (0.014)	0.37 (0.047)	0.25 (0.002)	0.28 (0.025)	0.21 (0.009)	0.24 (0.028)	0.17 (0.002)	0.20 (0.013)
	F&F	0.36 (0.026)	0.40 (0.053)	0.27 (0.002)	0.31 (0.026)	0.22 (0.003)	0.25 (0.033)	0.19 (0.001)	0.21 (0.017)
	M&M	0.36 (0.002)	0.38 (0.048)	0.26 (0.002)	0.29 (0.026)	0.22 (0.012)	0.24 (0.032)	0.18 (0.002)	0.20 (0.015)
GSP, SBA, cortical thickness	F&M	0.37 (0.003)	0.39 (0.027)	0.28 (0.018)	0.32 (0.040)	0.23 (0.009)	0.24 (0.014)	0.18 (0.017)	0.20 (0.017)
	F&F	0.37 (0.001)	0.39 (0.038)	0.29 (0.009)	0.32 (0.053)	0.23 (0.002)	0.24 (0.009)	0.19 (0.008)	0.21 (0.013)
	M&M	0.37 (0.005)	0.40 (0.022)	0.29 (0.015)	0.33 (0.035)	0.23 (0.011)	0.24 (0.024)	0.20 (0.013)	0.21 (0.018)
GSP, SBA, volume	F&M	0.24 (0.002)	0.27 (0.059)	0.18 (0.003)	0.19 (0.026)	0.14 (0.002)	0.16 (0.010)	0.12 (0.012)	0.15 (0.011)
	F&F	0.48 (0.004)	0.46 (0.035)	0.36 (0.002)	0.42 (0.032)	0.31 (0.005)	0.34 (0.038)	0.26 (0.005)	0.30 (0.023)
	M&M	0.42 (0.001)	0.47 (0.032)	0.33 (0.002)	0.33 (0.042)	0.28 (0.003)	0.29 (0.027)	0.24 (0.008)	0.25 (0.029)
GSP, SBA, “corrected” volume	F&M	0.38 (0.032)	0.40 (0.045)	0.27 (0.003)	0.31 (0.029)	0.22 (0.011)	0.24 (0.013)	0.19 (0.008)	0.21 (0.022)
	F&F	0.36 (0.021)	0.38 (0.034)	0.26 (0.003)	0.30 (0.025)	0.22 (0.009)	0.23 (0.013)	0.18 (0.006)	0.21 (0.020)
	M&M	0.41 (0.048)	0.43 (0.059)	0.30 (0.007)	0.34 (0.033)	0.24 (0.014)	0.27 (0.018)	0.20 (0.010)	0.23 (0.027)

		**Mean (SD) chances to be in the same cluster, for 7–10 divisions**							

**Number of clusters:**		**7**		**8**		**9**		**10**	
		**K**	**H**	**K**	**H**	**K**	**H**	**K**	**H**

Connectomes+ VBM	F&M	0.17 (0.010)	0.17 (0.019)	0.15 (0.005)	0.16 (0.020)	0.13 (0.007)	0.14 (0.011)	0.11 (0.007)	0.13 (0.013)
	F&F	0.18 (0.019)	0.19 (0.019)	0.16 (0.006)	0.18 (0.021)	0.14 (0.007)	0.15 (0.010)	0.13 (0.007)	0.15 (0.011)
	M&M	0.17 (0.019)	0.17 (0.019)	0.14 (0.003)	0.16 (0.020)	0.13 (0.009)	0.13 (0.009)	0.11 (0.004)	0.13 (0.011)
GSP VBM	F&M	0.15 (0.004)	0.17 (0.013)	0.13 (0.007)	0.15 (0.014)	0.12 (0.008)	0.11 (0.008)	0.11 (0.006)	0.12 (0.013)
	F&F	0.17 (0.005)	0.19 (0.011)	0.14 (0.008)	0.16 (0.012)	0.13 (0.006)	0.12 (0.011)	0.12 (0.006)	0.13 (0.016)
	M&M	0.16 (0.004)	0.18 (0.015)	0.14 (0.007)	0.16 (0.018)	0.13 (0.008)	0.11 (0.006)	0.11 (0.007)	0.12 (0.012)
GSP, SBA, cortical thickness	F&M	0.16 (0.016)	0.18 (0.018)	0.14 (0.019)	0.16 (0.027)	0.12 (0.014)	0.14 (0.020)	0.11 (0.015)	0.12 (0.013)
	F&F	0.17 (0.003)	0.19 (0.011)	0.15 (0.010)	0.17 (0.018)	0.13 (0.007)	0.15 (0.012)	0.12 (0.008)	0.13 (0.010)
	M&M	0.18 (0.009)	0.19 (0.018)	0.15 (0.012)	0.17 (0.028)	0.14 (0.008)	0.15 (0.020)	0.12 (0.012)	0.13 (0.010)
GSP, SBA, volume	F&M	0.10 (0.007)	0.11 (0.012)	0.09 (0.004)	0.09 (0.007)	0.08 (0.007)	0.09 (0.007)	0.07 (0.004)	0.08 (0.009)
	F&F	0.22 (0.002)	0.24 (0.019)	0.20 (0.013)	0.21 (0.028)	0.19 (0.015)	0.20 (0.021)	0.16 (0.003)	0.17 (0.008)
	M&M	0.21 (0.005)	0.21 (0.020)	0.18 (0.015)	0.18 (0.010)	0.15 (0.014)	0.16 (0.013)	0.14 (0.009)	0.16 (0.017)
GSP, SBA, “corrected” volume	F&M	0.16 (0.007)	0.18 (0.015)	0.14 (0.005)	0.15 (0.017)	0.12 (0.004)	0.14 (0.013)	0.11 (0.004)	0.12 (0.004)
	F&F	0.15 (0.006)	0.17 (0.010)	0.13 (0.003)	0.15 (0.012)	0.12 (0.004)	0.13 (0.012)	0.11 (0.004)	0.12 (0.003)
	M&M	0.17 (0.011)	0.20 (0.020)	0.15 (0.008)	0.17 (0.022)	0.13 (0.005)	0.15 (0.015)	0.12 (0.005)	0.14 (0.006)

*F, female; H, hierarchical; K, *k*-means; M, male.*

Analysis of the GSP-VBM and GSP-cortical thickness datasets revealed results very similar to those obtained with the Connectomes+ dataset, with *P* stabilizing around 0.56 and 0.53, respectively (**Figures 3C,D, 4C,D**), and very similar chances that a female and a male would be in the same cluster and that two males or two females would be in the same cluster (**Table 2**). In contrast, analysis of the GSP “uncorrected” volumes revealed a different pattern of results: Large sex differences existed regardless of cluster size, as reflected in mean *P* around 0.70 and large variability of *P* (**Figures 3E, 4E**), and the chances that a female and a male would be in the same cluster were about half the chances that two males or two females would be in the same cluster (**Table 2**). As with the other analyses, analysis of the same dataset following “correction” for brain size revealed a pattern of results very similar to the one obtained with the VBM and cortical thickness datasets, with *P* stabilizing around 0.52 (**Figures 3F, 4F**) and very similar chances that a female and a male would be in the same cluster and that two males or two females would be in the same cluster (**Table 2**).

### Supervised Clustering: Are the Brain Types Typical of Females and Males in One Subpopulation Also Typical of Females and Males in Other Subpopulations?

**FIGURE 5:**
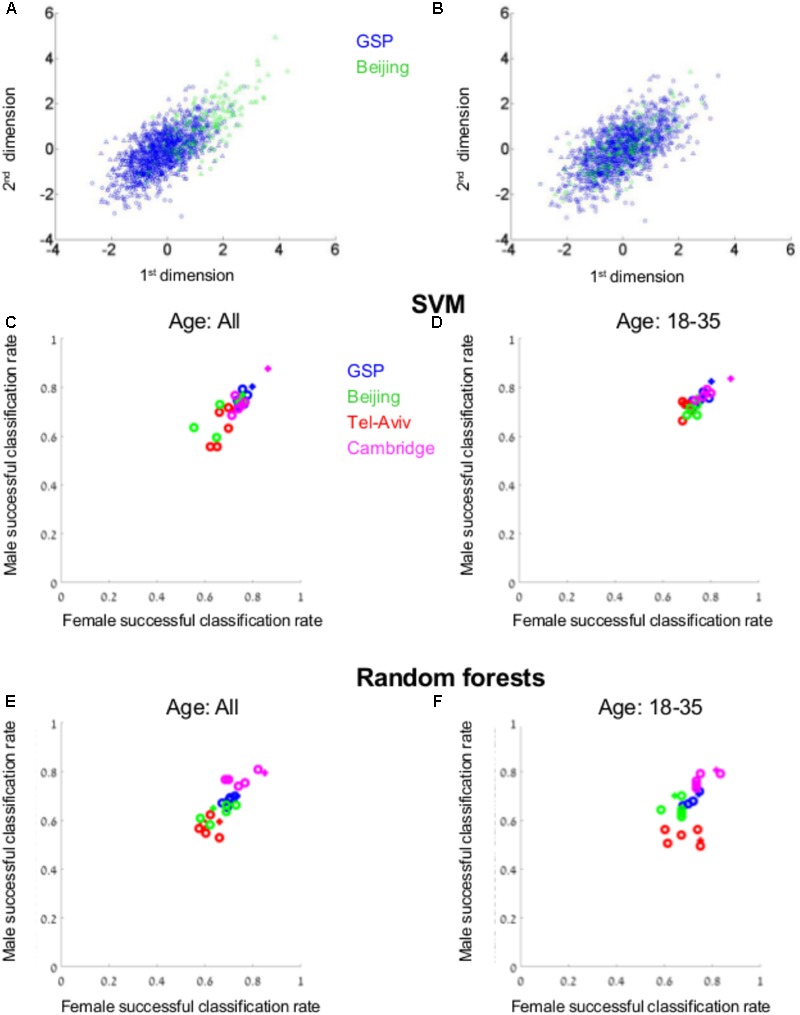
Supervised clustering. **(A)** The distribution of brains from females (circles) and from males (triangles) from the GSP (blue) and Beijing (green) datasets following *z*-score transformation of the combined GSP and Beijing datasets **(B)** and when each dataset was transferred to *z*-scores prior to transferring the combined dataset into *z*-scores. **(C–F)** The percent of females (out of females, *X*-axis) and the percent of males (out of males, *Y*-axis) that were correctly classified as female or male, respectively, by a model created by SVM **(C,D)** or random forests **(E,F)** on the GSP VBM data, when the model was tested on the entire test dataset **(C,E)** or only on participants aged 18–35 years **(D,F)**. The classification rates for the GSP data (blue dots) were calculated using 10-folds cross-validation. The classification rates for Tel-Aviv, Cambridge, and Beijing are marked in green, purple, and red, respectively. The *z*-scores transformation is marked with a plus.

For this analysis we used the VBM data of four subpopulations, each from a different geographical region – Boston (the GSP dataset), Tel-Aviv, Cambridge, and Beijing. As has previously been reported (e.g., Lee et al., 2005; Tang et al., 2010), there were differences between the four datasets in their spread over space (e.g., **Figure 5A**). Therefore, each dataset was first transformed to *z*-scores, to assure that all samples share the same center and spread (compare, e.g., **Figures 5A,B**). SVM was then applied to the GSP-VBM dataset, which was the largest among the VBM datasets from a single geographical region. Applying 10-folds cross-validation to the six transformations of the GSP-VBM dataset (blue dots in **Figure 5C**), the classification rates varied between 72 and 82% (average = 76.5%) for males (*Y*-axis), and between 72 and 80% (average = 76.6%) for females (*X*-axis), depending on the data transformation method (the plus marks the *z*-score transformation). Under the best separation, the chances for a male and a female to be in the same cluster were 32%, compared with 68 and 68% for two females and two males, respectively.

We next tested whether the model created to best separate between brains from females and males in the GSP-VBM dataset similarly separates brains from females and males in datasets obtained in Tel-Aviv (red), Cambridge (purple), and Beijing (green). For each test dataset, following dimension reduction on the combined GSP and test dataset, a model was built on the GSP data, and then the classification rate for the test dataset was calculated using this model, both for the entire test dataset (**Figure 5C**) and for a subset of individuals in the same age range as in the GSP-VBM dataset (18–35 years old, **Figure 5D**). Whereas accuracy rates for the Cambridge sample were similar to those for the GSP sample, they were lower for the Beijing and Tel-Aviv samples.

Last, we applied SVM (using 10-folds cross-validation) to each of the Cambridge, Beijing, and Tel-Aviv samples, and compared the classification of each brain using these models, to the classification according to the model created using the GSP dataset. We found that the percent of brains that were similarly classified by the GSP model and by a model created on the test sample was often not statistically different from the percent expected if the two models were not related, but very different from the percent expected if the “male” and “female” clusters created by the two models were completely overlapping (**Table 3**).

**Table 3:** Comparing models created by the supervised clustering algorithms on different samples (ages 18–35 years only).

SVM															
	**Tel Aviv**					**Beijing**					**Cambridge**				
	**Self**	**GSP**	**U**	**P**	**A**	**Self**	**GSP**	**U**	**P**	**A**	**Self**	**GSP**	**U**	**P**	**A**

*z*-scores	53	71	51	82	47^ns,2^	73	75	61	98	71^ns,2^	66	86	62	80	74^ns,ns^
PCA	59	72	54	86	59^ns,2^	74	69	59	95	75^1,2^	79	73	63	93	77^1,2^
DM	72	69	59	98	67^ns,2^	64	75	57	89	69^ns,2^	70	78	61	92	76^2,2^
μIDM	73	70	60	97	69^ns,2^	66	75	58	91	69^ns,2^	73	78	63	95	78^2,2^
ICPQR	74	64	57	90	64^ns,2^	63	59	53	96	66^1,2^	77	74	63	97	83^2,2^
ICPQR	54	62	51	93	51^ns,2^	70	61	54	91	62^ns,2^	78	71	62	93	76^1,2^
**Random forests**															
	**Tel Aviv**					**Beijing**					**Cambridge**				
	**Self**	**GSP**	**U**	**P**	**A**	**Self**	**GSP**	**U**	**P**	**A**	**Self**	**GSP**	**U**	**P**	**A**

*z*-scores	78	63	58	85	65^ns,2^	64	67	55	97	79^2,2^	72	81	63	91	83^2,ns^
PCA	70	58	54	88	58^ns,2^	66	64	55	98	69^ns,2^	73	77	63	96	76^ns,2^
DM	70	65	56	92	55^ns,2^	58	69	53	89	62^ns,2^	64	81	58	82	69^ns,ns^
μIDM	72	56	53	84	53^ns,2^	60	66	53	94	59^ns,2^	70	73	59	97	70^ns,2^
ICPQR	70	60	53	86	61^ns,2^	56	61	51	95	64^1,2^	65	74	57	91	76^1,1^
ICPQR	69	62	54	85	58^ns,2^	69	65	56	94	66^ns,2^	71	75	60	96	72^ns,2^

*Self, the percent of brains from the test sample that were correctly classified by a model created on the test sample (applying 10-folds cross-validation); GSP, the percent of brains from the test sample that were correctly classified by a model created on the GSP sample; U, the percent of brains expected to be similarly classified by the GSP and Self models if they were unrelated; P, the percent of brains expected to be similarly classified by the GSP and Self models if they were perfectly overlapping; A, the actual percent of brains similarly classified by the GSP and Self models; the uppercase text marks whether this percentage was significantly different from the percent expected if the models were unrelated and perfectly overlapping, respectively. ns, not significant; 1, *p* < 0.0014 (alpha following the Dunn–Šidák correction for multiple comparisons); 2, *p* < 0.0001; PCA, principle component analysis; DM, diffusion mapping, Euclidean distances; μIDM, isometric diffusion map; ICPQR, incomplete pivoted QR decomposition with the kernel of the diffusion map; ICPQRd, incomplete pivoted QR decomposition without the kernel of the diffusion map.*

Repeating these analyses using another supervised clustering algorithm, random forests, yielded a similar pattern of results: Classification rates of between 65 and 70% (average = 68%) for males, and between 67 and 73% (average = 71%) for females from the GSP dataset (**Figure 5E**); applying the GSP model to the test samples yielded higher accuracy rates for the Cambridge sample, but lower rates for the Tel-Aviv and Beijing samples (**Figures 5E,F**); and comparing the classification by the GSP model to the classification of a model created on each test dataset revealed that the percent of brains that were similarly classified by the two models was often not statistically different from the percent expected if the two models were not related, but very different from the percent expected if the “male” and “female” clusters created by the two models were completely overlapping (**Table 3**).

## Discussion

The results of the various approaches applied in the present study are at variance with the belief that the effects of sex on brain structure “add up” to create two types of brain, one typical of females and the other typical of males. Rather, they support the claim that the types of brain typical of females are also typical of males, and that large sex differences exist in the prevalence of some rare brain types (Joel, 2011, 2012; Joel et al., 2015; Joel and Fausto-Sterling, 2016).

Specifically, the anomaly detection analysis revealed that regardless of sample, type of analysis of the MR images (volume- and surface-based), type of data (“corrected” volume of gray matter regions extracted by VBM, “corrected” volume of gray and white matter regions, and “uncorrected” cortical thickness extracted by FreeSurfer), and type of dimension reduction, the forms of brain typical of females were also typical of males, and vice versa. In contrast, when the “uncorrected” volume of gray and white matter regions was considered, the anomaly detection algorithm could better differentiate between brains from females and brains from males. Note, however, that this better detection rate (1.68 and 3.05, compared to <1.25 for the other datasets) was still much lower than that obtained for the gender-stereotyped behaviors (>22). In addition, this better separation was attributed to the difference between females and males in total brain volume.

Applying unsupervised clustering algorithms to divide brains into two clusters revealed that regardless of sample, type of analysis of the MR images (except for the “uncorrected” volumes, see below), type of data, type of dimension reduction, and clustering algorithm, the proportions of males and females in the large cluster were similar (even when clusters were of comparable size). Whether the brain “type” most common in females was also the brain “type” most common in males, depended, however, on the sample and clustering algorithm. In two datasets (Connectomes+ and GSP “corrected” volume), this description was preferred by the two algorithms, whereas in the remaining two datasets (GSP-VBM and GSP-cortical thickness), this description was almost always preferred by the hierarchical algorithm, but not by the *k*-means algorithm. Yet, even in cases where the majority of females were in one cluster and the majority of males in the other, the proportions of males and females in each cluster were similar, so that in all cases a female and a male were almost as likely to have the same brain type (i.e., be in the same cluster) as two females or two males. This was also true when brains were separated into a larger number of clusters (3–10). This latter analysis further revealed that large sex differences in the proportion of females and males in a cluster might exist, but only in small clusters (typically of size less than 150 brains). This result supports Joel’s hypothesis that whereas the forms of brain typical of females are also typical of males and vice versa, there are sex differences in the prevalence of some rare brain types.

Only when unsupervised clustering was applied to the “uncorrected” volume of gray and white matter regions, brains from females and males were more separated, with the chance that two males or two females would be in the same cluster being about twice the chance that a female and a male would be in the same cluster. It is noteworthy that this ratio, while higher than that obtained when unsupervised clustering algorithms were applied to “uncorrected” cortical thickness or to “corrected” volume of gray and white matter regions (∼1.1), is much lower than the ratio obtained by these algorithms when applied to the gender-stereotyped behavior (>6). Moreover, as was the case for the anomaly detection analysis, the better separation achieved for the “uncorrected” volume of gray and white matter regions was attributed to the differences between females and males in total brain volume.

The present finding that the main morphological difference between brains from females and from males is in total brain volume is in line with previous reports that most sex differences in the morphology of specific brain structures disappear or become trivial when total brain volume is factored out (e.g., Im et al., 2008; Hänggi et al., 2014; Jäncke et al., 2015; Coupe et al., 2017). We leave the question of whether brain volume is directly controlled by sex-specific influences or is a by-product of sex differences in body size, to others. Yet, what our study shows is that human females and males are highly similar in brain architecture, that is, in the relations between the size of different brain structures, with brain architectures common in one sex also common in the other, and large sex differences existing only in the frequency of some rare brain architectures.

As expected, a supervised clustering algorithm achieved better separation between brains from females and brains from males than the unsupervised algorithms. Thus, applying SVM to the “corrected” volume of gray matter regions, the best separation achieved for the GSP-VBM dataset was 80.4% of the males in one cluster and 78% of the females in the other cluster (compared to 57 and 62%, respectively, which was the best separation achieved for this dataset with an unsupervised clustering algorithm). With this classification rate, which is similar to those obtained by others for similar datasets (Chekroud et al., 2016; Del Giudice et al., 2016; Rosenblatt, 2016), the chances that two males or two females would be in the same cluster (68%) were about twice the chances that a female and a male would be in the same cluster (32%) – higher than that obtained when unsupervised clustering algorithms were applied to this dataset, but much lower than the ratio obtained by these unsupervised algorithms when applied to gender-stereotyped behaviors. Moreover, the classification rates obtained by the supervised algorithm were lower when the models created on the GSP-VBM dataset were tested on some of the other datasets, and, most importantly, the “male” and “female” clusters of the GSP models often did not match the “male” and “female” clusters of models created on the test dataset. In other words, even after “correcting” for inter-sample differences in gross measures of the brain, there were inter-sample differences in the spread of brains from females and from males, so that brain types that were considered typical of one sex category in one sample were sometimes considered typical of the other sex category in another sample. Although the present study cannot reveal the cause of these inter-sample differences, their existence does not support a universal division into a female-typical and a male-typical brain structure.

**FIGURE 6:**
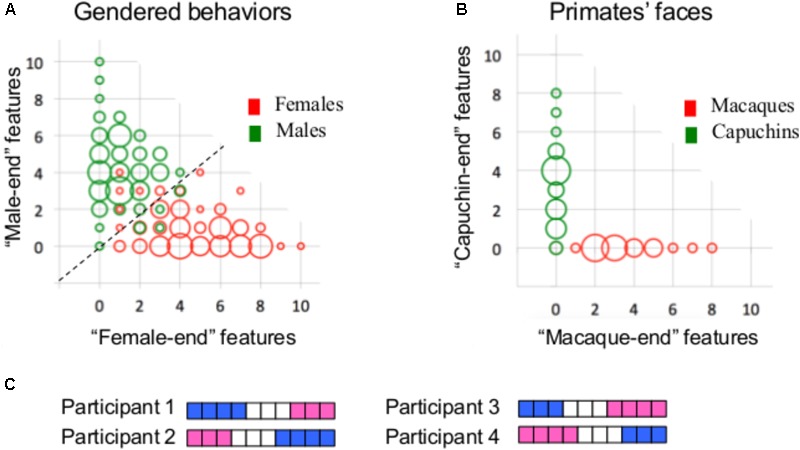
**(A)** A bivariate scattergram of the number of features at the “female-end” (*X-*axis) and at the “male-end” (*Y*-axis) in females (red) and males (green) in Carothers and Reis’ gender-stereotyped behavioral dataset. Using the actual distributions of males and females in the sample, a “male-end” and a “female-end” zones were arbitrarily defined as the scores of the 33% most extreme males and females, respectively, and an “intermediate” zone was defined as the area in-between these two (adapted from Joel et al., 2015). **(B)** Same as **A**, but for the 10 facial morphology features showing the largest differences between the two primate species. **(C)** An illustration of the gender mosaic in four hypothetical participants. Scores on the 10 gender stereotyped behaviors are represented using a pink–white–blue (“female-end”–“intermediate”–“male-end”) color code. Each horizontal line represents a single participant and each column represents a single behavior.

The present conclusion that the brain types typical of females are also typical of males, and vice versa, is restricted to the specific analytical approaches that were used in the present study. Clearly, it is possible that different results would be obtained using other approaches or other brain-related datasets. We would like to stress, however, that in our view, the finding that most human brains are comprised of unique mosaics of features, some more common in females, some more common in males, and some similarly common in females and males, is sufficient for concluding that human brains do not belong to two types, and it is not necessary to further demonstrate that there is no mathematical sense in which brains from females and from males are separable. This point can be made clear by contrasting the primate facial morphology data with the gender-stereotyped behavior data, which are both separable into two (more or less) distinct types using mathematical tools, but show opposite patterns of mosaicism. Thus, whereas the number of participants with a mosaic of gender-stereotyped behaviors is much higher than the number of participants who show internal consistency in their gender characteristics [i.e., they have either only “female-end” (i.e., more common in females compared to males) or only “male-end” (i.e., more common in males compared to females) characteristics, **Figure 6A**], the reverse is true for the primate facial morphology data (**Figure 6B**, see also Del Giudice et al., 2016). We suggest that the interpretation of these results should be that the primate facial morphology data come from two distinct populations (as is indeed the case) whereas the human gender-stereotyped behaviors do not, because the high degree of mosaicism makes the division of humans into two clusters functionally meaningless, even though it is possible mathematically. Consider, for example, a division of humans into two clusters or types on the basis of the number of “female-end” and “male-end” characteristics – one type, characterized by more “male-end” than “female-end” characteristics, would contain 99% of the males and 14% of the females in Carothers and Reis’ sample, whereas the other type, characterized by more (or the same) number of “female-end” than “male-end” characteristics, would contain 86% of the females and 1% of the males. Thus, a person’s sex category can be used to quite accurately predict whether s/he will have more “female-end” or more “male-end” characteristics. However, one’s sex category does not provide information on the number of “female-end” and “male-end” characteristics nor on which characteristics are “female-end” and which are “male-end.” Yet, it is this latter type of information that defines a person’s character or behavior. Consider for example two hypothetical participants, Participant 1 and Participant 2, whose gender mosaic is presented in **Figure 6C**. Both have four “male-end” characteristics (in blue) and three “female-end” characteristics (in pink), and thus belong to the same type of humans. However, in terms of the actual composition of their gender characteristics, they are almost as different as two participants can be. In contrast, Participant 1 is very similar to Participant 3, and Participant 2 is very similar to Participant 4, even though Participants 3 and 4, which have three “male-end” and four “female-end” characteristics, belong to the second type of humans.

Last, we would like to stress that the present demonstration that brain architectures common in females are also common in males and that large sex differences exist only in the frequency of some rare brain architectures, cannot be directly linked to similarities and differences between males and females in behavior or in susceptibility to pathology. This is because, as discussed above, the present analytical methods may have grouped together brains that differ widely morphologically, and because brain morphology cannot directly be linked to normal and abnormal behavior (De Vries, 2004; de Vries and Södersten, 2009).

## Conclusion

We have recently discovered that most human brains are composed of unique mosaics of features, and concluded that human brains do not belong to two distinct types, “male” and “female,” and that one’s sex category provides very little information about the specific composition of one’s unique brain mosaic (Joel et al., 2015). The present study supports these conclusions by showing that even when biological relevance is ignored, the structure of human brains does not fit into two distinct types of brain, one typical of males and the other typical of females. Moreover, although it is possible to use one’s brain architecture to predict whether this person is female or male with accuracy of ∼80%, one’s sex category provides very little information on the likelihood that one’s brain architecture is similar to or different from someone else’s brain architecture. This is because the brain types typical of females are also typical of males, and large sex differences are found only in the prevalence of some rare brain types.

It follows that whereas both female and male participants should be used in every study of the structure and function of the human brain to better represent the entire variability of our species, the use of sex category as a variable in analyzing the results of such studies should not be the default. This is because in studies of the typical human brain (as opposed to studies of rare conditions, such as autism, schizophrenia, etc.) using sex category as a variable would not control for sex category-related variability but rather lead to the detection of chance differences between the groups of females and males in the study (Joel, 2011; Joel and Fausto-Sterling, 2016; Joel and McCarthy, 2016). Evidence supporting this claim has been published recently (David et al., 2018).

On the basis of the present and our previous study (Joel et al., 2015), we suggest that detection of differences between females and males in a given system (e.g., the brain, the immune system) should not be unconditionally interpreted as indicating that there is one form of the system which is typical of males, and another, typical of females. Nor should such a conclusion be based on the ability to mathematically divide the data into two clusters, one including mostly females and the other including mostly males. Rather, to conclude that a system comes in two forms, one typical of females and the other typical of males, one needs to demonstrate that internal consistency is much more prevalent than mosaicism (Joel et al., 2015, 2016). Regarding the brain, our previous and present findings call for a shift in our conceptualization of the relations between sex and the brain from dimorphism to mosaic, and for the development of analytical methods that take into account the variability in the human brain (rather than treat it as noise) as well as individual differences in the specific composition of the brain mosaic. Analytical methods with the above characteristics have been developed for working with other types of data. Specifically, with the explosion of large-scale biological data following the sequencing of the human genome, methods for analysis of large-scale gene expression data have been developed and used for detecting patterns of change that are characteristic of specific disorders. In parallel, the understanding that genes and proteins do not work in isolation has led to the development of methods for describing how genes/proteins work in a network. Given that information processing in the brain also depends on networks that are comprised of many brain regions rather than on regions working in isolation, we believe that such methods are necessary also for studying the relation between brain structure and (dys)function.

## Ethics Statement

The study used existing data sets of human brain images.

## Author Contributions

DJ framed the research question. DJ, AP, MS, IM, and AA adapted existing analytic tools to the research question and wrote the paper. IM developed new analytic tools. ZB and SO prepared the data for analysis. AP performed the research.

## Conflict of Interest Statement

The authors declare that the research was conducted in the absence of any commercial or financial relationships that could be construed as a potential conflict of interest.

## Footnotes

1 https://github.com/human-brain/article-code

2 http://www.fil.ion.ucl.ac.uk/spm

3 http://surfer.nmr.mgh.harvard.edu/fswiki

4 https://github.com/human-brain/article-code
