# Supplementary material for: Analysis of Human Brain Structure Reveals that the Brain “Types” Typical of Males Are Also Typical of Females, and Vice Versa
Source: Front Hum Neurosci. 2018 Oct 18;12:399. doi: 10.3389/fnhum.2018.00399 (PMC6204758; doi:10.3389/fnhum.2018.00399)
Supplement: Supplementary file 1 [file Data_Sheet_1.docx]

# Appendix I: Labels of brain regions used in the Volume-based (VBM) analyses (Automated Anatomical Labeling (AAL) Atlas)

| Left precentral gyrus |
| --- |
| Right precentral gyrus |
| Left superior frontal gyrus, dorsolateral |
| Right superior frontal gyrus, dorsolateral |
| Left superior frontal gyrus, orbital part |
| Right superior frontal gyrus, orbital part |
| Left middle frontal gyrus, lateral part |
| Right middle frontal gyrus, lateral part |
| Left middle frontal gyrus, orbital part |
| Right middle frontal gyrus, orbital part |
| Left opercular part of inferior frontal gyrus |
| Right opercular part of inferior frontal gyrus |
| Left area triangularis |
| Right area triangularis |
| Left orbital part of inferior frontal gyrus |
| Right orbital part of inferior frontal gyrus |
| Left rolandic operculum |
| Right rolandic operculum |
| Left supplementary motor area |
| Right supplementary motor area |
| Left olfactory cortex |
| Right olfactory cortex |
| Left superior frontal gyrus, medial part |
| Right superior frontal gyrus, medial part |
| Left superior frontal gyrus, medial orbital part |
| Right superior frontal gyrus, medial orbital part |
| Left gyrus rectus |
| Right gyrus rectus |
| Left insula |
| Right insula |
| Left anterior cingulate gyrus |
| Right anterior cingulate gyrus |
| Left middle cingulate |
| Right middle cingulate |
| Left posterior cingulate gyrus |
| Right posterior cingulate gyrus |
| Left hippocampus |
| Right hippocampus |
| Left parahippocampal gyrus |
| Right parahippocampal gyrus |
| Left amygdala |
| Right amygdala |
| Left calcarine sulcus |
| Right calcarine sulcus |
| Left cuneus |
| Right cuneus |
| Left lingual gyrus |
| Right lingual gyrus |
| Left superior occipital |
| Right superior occipital |
| Left middle occipital |
| Right middle occipital |
| Left inferior occipital |
| Right inferior occipital |
| Left fusiform gyrus |
| Right fusiform gyrus |
| Left postcentral gyrus |
| Right postcentral gyrus |
| Left superior parietal lobule |
| Right superior parietal lobule |
| Left inferior parietal lobule |
| Right inferior parietal lobule |
| Left supramarginal gyrus |
| Right supramarginal gyrus |
| Left angular gyrus |
| Right angular gyrus |
| Left precuneus |
| Right precuneus |
| Left paracentral lobule |
| Right paracentral lobule |
| Left caudate nucleus |
| Right caudate nucleus |
| Left putamen |
| Right putamen |
| Left globus pallidus |
| Right globus pallidus |
| Left thalamus |
| Right thalamus |
| Left transverse temporal gyri |
| Right transverse temporal gyri |
| Left superior temporal gyrus |
| Right superior temporal gyrus |
| Left superior temporal pole |
| Right superior temporal pole |
| Left middle temporal gyrus |
| Right middle temporal gyrus |
| Left middle temporal pole |
| Right middle temporal pole |
| Left inferior temporal gyrus |
| Right inferior temporal gyrus |
| Left crus I of cerebellar hemisphere |
| Right crus I of cerebellar hemisphere |
| Left crus II of cerebellar hemisphere |
| Right crus II of cerebellar hemisphere |
| Left Lobule III of cerebellar hemisphere |
| Right Lobule III of cerebellar hemisphere |
| Left lobule IV, V of cerebellar hemisphere |
| Right lobule IV, V of cerebellar hemisphere |
| Left Lobule VI of cerebellar hemisphere |
| Right Lobule VI of cerebellar hemisphere |
| Left lobule VIIB of cerebellar hemisphere |
| Right lobule VIIB of cerebellar hemisphere |
| Left lobule VIII of cerebellar hemisphere |
| Right lobule VIII of cerebellar hemisphere |
| Left lobule IX of cerebellar hemisphere |
| Right lobule IX of cerebellar hemisphere |
| Left lobule X of cerebellar hemisphere (flocculus) |
| Right lobule X of cerebellar hemisphere (flocculus) |
| Lobule I, II of vermis |
| Lobule III of vermis |
| Lobule IV, V of vermis |
| Lobule VI of vermis |
| Lobule VII of vermis |
| Lobule VIII of vermis |
| Lobule IX of vermis |
| Lobule X of vermis (nodulus) |

# Appendix II: Labels of brain regions used in the Surface-based analyses (cortical thickness and volume) (Desikan-Killiany Atlas).

| Brain region | Volume | Cortical thickness |
| --- | --- | --- |
| rh_bankssts | +* | + |
| rh_caudalanteriorcingulate | +* | + |
| rh_caudalmiddlefrontal | +* | + |
| rh_cuneus | +* | + |
| rh_entorhinal | +* | + |
| rh_fusiform | +* | + |
| rh_inferiorparietal | +* | + |
| rh_inferiortemporal | +* | + |
| rh_isthmuscingulate | +* | + |
| rh_lateraloccipital | +* | + |
| rh_lateralorbitofrontal | +* | + |
| rh_lingual | +* | + |
| rh_medialorbitofrontal | +* | + |
| rh_middletemporal | +* | + |
| rh_parahippocampal | +* | + |
| rh_paracentral | +* | + |
| rh_parsopercularis | +* | + |
| rh_parsorbitalis | +* | + |
| rh_parstriangularis | +* | + |
| rh_pericalcarine | +* | + |
| rh_postcentral | +* | + |
| rh_posteriorcingulate | +* | + |
| rh_precentral | +* | + |
| rh_precuneus | +* | + |
| rh_rostralanteriorcingulate | +* | + |
| rh_rostralmiddlefrontal | +* | + |
| rh_superiorfrontal | +* | + |
| rh_superiorparietal | +* | + |
| rh_superiortemporal | +* | + |
| rh_supramarginal | +* | + |
| rh_frontalpole | +* | + |
| rh_temporalpole | +* | + |
| rh_transversetemporal | +* | + |
| rh_insula | +* | + |
| lh_bankssts | +* | + |
| lh_caudalanteriorcingulate | +* | + |
| lh_caudalmiddlefrontal | +* | + |
| lh_cuneus | +* | + |
| lh_entolhinal | +* | + |
| lh_fusiform | +* | + |
| lh_inferiorparietal | +* | + |
| lh_inferiortemporal | +* | + |
| lh_isthmuscingulate | +* | + |
| lh_lateraloccipital | +* | + |
| lh_lateralorbitofrontal | +* | + |
| lh_lingual | +* | + |
| lh_medialorbitofrontal | +* | + |
| lh_middletemporal | +* | + |
| lh_parahippocampal | +* | + |
| lh_paracentral | +* | + |
| lh_parsopercularis | +* | + |
| lh_parsorbitalis | +* | + |
| lh_parstriangularis | +* | + |
| lh_pericalcarine | +* | + |
| lh_postcentral | +* | + |
| lh_posteriorcingulate | +* | + |
| lh_precentral | +* | + |
| lh_precuneus | +* | + |
| lh_rostralanteriorcingulate | +* | + |
| lh_rostralmiddlefrontal | +* | + |
| lh_superiorfrontal | +* | + |
| lh_superiorparietal | +* | + |
| lh_superiortemporal | +* | + |
| lh_supramarginal | +* | + |
| lh_frontalpole | +* | + |
| lh_temporalpole | +* | + |
| lh_transversetemporal | +* | + |
| lh_insula | +* | + |
| Left-UnsegmentedWhiteMatter | + |  |
| Right-UnsegmentedWhiteMatter | + |  |
| lhCorticalWhiteMatterVol | + |  |
| rhCorticalWhiteMatterVol | + |  |
| CorticalWhiteMatterVol | + |  |
| MaskVol | + |  |
| EstimatedTotalIntraCranialVol | + |  |
| Left-Lateral-Ventricle | + |  |
| Left-Inf-Lat-Vent | + |  |
| Left-Cerebellum-White-Matter | + |  |
| Left-Cerebellum-Cortex | + |  |
| Left-Thalamus-Proper | + |  |
| Left-Caudate | + |  |
| Left-Putamen | + |  |
| Left-Pallidum | + |  |
| 3rd-Ventricle | + |  |
| 4th-Ventricle | + |  |
| Brain-Stem | + |  |
| Left-Hippocampus | + |  |
| Left-Amygdala | + |  |
| CSF | + |  |
| Left-Accumbens-area | + |  |
| Left-VentralDC | + |  |
| Left-vessel | + |  |
| Left-choroid-plexus | + |  |
| Right-Lateral-Ventricle | + |  |
| Right-Inf-Lat-Vent | + |  |
| Right-Cerebellum-White-Matter | + |  |
| Right-Cerebellum-Cortex | + |  |

* The volume analysis included the volume of this cortical region as well as the volume of the white matter region beneath it.

# Appendix III

Data for this analysis consists of clusters obtained by various clustering methods and targeted number of clusters. Each such cluster provides two numerical features, cluster size n and sex disparity Y=max(X,n-X) where X is the female count in the cluster. The model paradigm is that for each cluster there is a latent random quantity 0 < Q < 1 such that, given that Q = q, X is binomially distributed with n trials and probability of success q. Let P = max(Q,1-Q). The model assumption is that there exist two smooth functions p(n) and s(n) such that a cluster of size n chooses P from a distribution with mean p(n) and standard deviation s(n). The purpose is to estimate the function p(.) taking into account the impact of the function s(.). The aim of this analysis was to test the hypothesis that p(.) tends to be a decreasing function fast approaching a constant level close to 0.5, and s(.) is relatively sizable only for small n. I.e., large clusters don’t differentiate sex and small clusters differentiate sex in a heterogeneous way.

A natural candidate for analysis is the disparity statistic Y/n-0.5 that in some way estimates the object of main interest p(n)-1/2. However, this statistic tends to be positively biased in a complicated way. A statistic more amenable to analysis is the quadratic disparity D=(Y/n-1/2)^2^, that can be expressed via the standardization of X as X = nq+√(nq(1-q))Z (with E[Z] = 0 and VAR[Z] = 1) in the form

D=(Y/n-1/2)^2^ = ((p-1/2)^2^ + 2(p-1/2)√(p(1-p)/n)Z+(p(1-p)/n)Z^2^

Since p(1-p)=1/4-(p-1/2)^2^, E[D|P=p] = 1/(4n)+((n-1)/n)(p-1/2)^2^. Hence, letting T = (n*D-1/4)/(n-1) be a conveniently chosen linear transformation of D, E[T|P=p] = (p-1/2)^2^.

The expectation E[T] is (E[P]-1/2)^2^ +VAR[P] but the extra variance term (of interest in its own right) turns out to be relatively small and may be ignored when estimating E[T]. In other words, T is essentially an unbiased estimator of (p-1/2)^2^. If E[T] can be estimated with small standard error, it can lead to a close estimator of p.

The proposed method of data analysis is as follows. Rank the clusters in increasing order of cluster size n and record for each cluster the value of T. This is a very noisy function but under the assumption that p(.) is smooth, p(.) can be estimated from the moving average of T in some window (plus or minus a fixed number of clusters) around each cluster size n. Similarly, s(.) can be estimated from the estimated p(.) in conjunction with the standard deviation of T in the moving window. The graphs can be embellished by applying smoothing splines or isotonic regression, but that would be contrary to the aim of letting data speak for itself.

The estimate ESTP of E[P] is 1/2 plus the square root of the moving average MAT(n) of T on the 100 clusters of size closest to each cluster size n. Let MVT(n) be the empirical moving variance of T in this interval. A formula was developed to obtain an estimator ESTV of s^2^(n) = VAR(P) with small bias provided s(n) is small enough.

ESTV = MVT(n)/(4*MAT(n)*(n/(n-1))^2^) – (1/4-MAT(n))/n

This formula, explained in the next paragraph, is incorporated into the MATLAB program that estimates the parameters as illustrated in Figure 4. As ESTV may come out negative, it is rather arbitrarily defined as 10^-6^ if smaller than this value.

Let ξ be the mean-zero noise in P = p + ξ. ESTV is the estimated variance of ξ. Express T as

T=n (p-1/2+ ξ +sqrt(p(1-p)/n)Z)^2^ -1/4)/(n-1)

~ (n(p-1/2)^2^(1+(2/(p-1/2))( ξ +√(p(1-p)/n)Z))-1/4)/(n-1)

which is a linear function of the sum ξ +√(p(1-p)/n)Z of two independent (small) random variables, with respective variances VAR[ξ] and p(1-p)/n. Hence,

VAR[T] = 4(n/(n-1))^2^(p-1/2)^2^(VAR[X]+(1/4-(p-1/2)^2^)/n).

In other words,

MVT(n) ~ 4(n/(n-1))^2^MAT(n)(VAR[ξ]+(1/4-MAT(n))/n)

from which VAR[ξ] can be extracted.
